# A case of endobronchial mucosa‐associated lymphoid tissue (MALT) lymphoma successfully treated with radiotherapy and a review of the literature

**DOI:** 10.1002/rcr2.1369

**Published:** 2024-05-08

**Authors:** Rino Arai, Mizuka Tanifuji, Asuka Nagai, Akinori Ebihara, Tokuzen Iwamoto, Shinnosuke Onaka, Mayumi Aoyama, Makoto Otaki, Hidenobu Shigemitsu, Ichiro Kuwahira

**Affiliations:** ^1^ Respiratory Disease Center Tokyo General Hospital Tokyo Japan; ^2^ Department of Diagnostic Imaging and Nuclear Medicine Tokyo Women's Medical University Tokyo Japan; ^3^ Department of Respiratory Medicine Niigata University School of Medicine Niigata Japan; ^4^ Department of Pulmonary Medicine Tokai University Tokyo Hospital Tokyo Japan; ^5^ Midtown Clinic Medical Corporation Himedic Yamanakako Yamanashi Japan; ^6^ Department of Critical Care Medicine St. Rose Dominican Siena Hospital Las Vegas Nevada USA

**Keywords:** endobronchial tumour, MALT lymphoma, radiation therapy, tracheal tumour

## Abstract

A 60‐year‐old man was noted to have an elevated lesion in the right mainstem bronchus on chest computed tomography (CT) during his annual medical checkup 3 years previously. The lesion had gradually increased in size. FDG‐PET showed no accumulation. Bronchoscopy revealed 5 nodular smooth surface protrusions on the ventral surface of the right mainstem bronchus, with the largest lesion that measured 5 mm in diameter. Biopsy showed diffuse infiltration of small lymphocytes, positive for CD20 and subsequently diagnosed with mucosa‐associated lymphoid tissue (MALT) lymphoma. The lesions disappeared on chest CT after radiotherapy, and no recurrence has been observed after 5 years. We reviewed 48 cases of endobronchial MALT lymphoma in the literature and provided a comprehensive review of the literature to date including our case.

## Graphical abstract

We herein report a rare case of endobronchial mucosa‐associated lymphoid tissue (MALT) lymphoma (EML) that was diagnosed through a chest computed tomography (CT) during an annual health checkup which was successfully treated with radiotherapy. To the best of our knowledge, only 48 cases of EML have been reported in the literature and are summarized here.

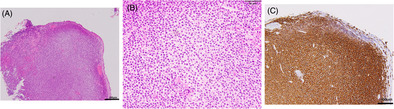

## INTRODUCTION

Endobronchial mucosa‐associated lymphoid tissue (MALT) lymphoma (EML) is a very rare disease and is not considered among the potential diagnoses when evaluating endobronchial lesions. We herein report a case of EML that was diagnosed through a chest computed tomography (CT) during an annual health checkup which was successfully treated with radiotherapy. To the best of our knowledge, only 48 cases of EML have been reported in the literature.^1^, ^2^, ^3^, ^4^, ^5^, ^6^, ^7^, ^8^, ^9^, ^10^, ^11^, ^12^, ^13^, ^14^, ^15^, ^16^, ^17^, ^18^, ^19^, ^20^, ^21^, ^22^, ^23^, ^24^, ^25^, ^26^, ^27^, ^28^, ^29^, ^30^, ^31^, ^32^, ^33^, ^34^, ^35^, ^36^, ^37^, ^38^ To date, a comprehensive review of this rare condition is not available. Therefore we believe reporting our case with a review of current literature will be useful for patients and providers who may encounter this disease in the future.

## CASE REPORT

**FIGURE 1:**
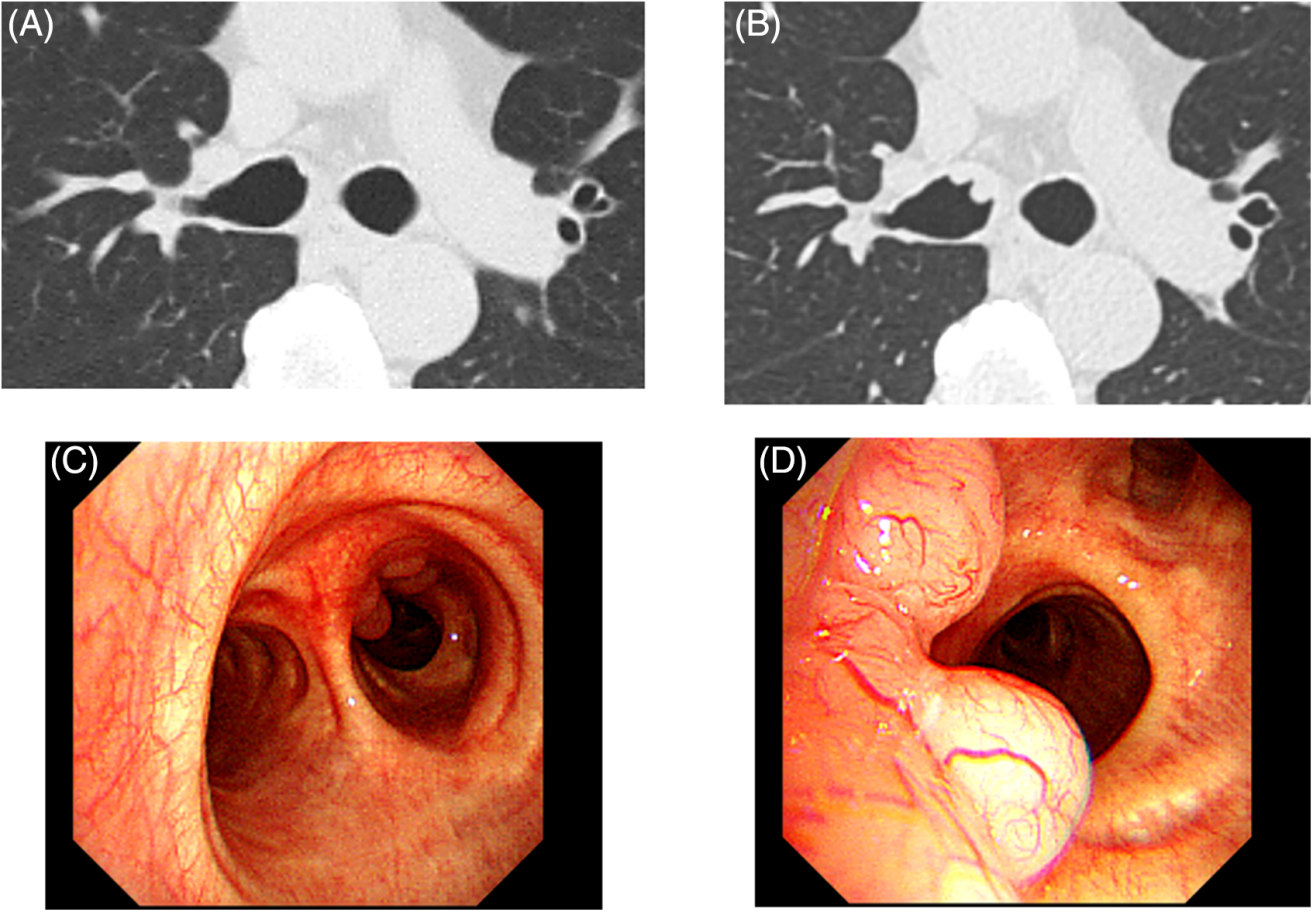
Chest CT (A, B) and Bronchoscopic findings (C, D). Chest CT showed multiple elevated lesions on the ventral side of the right main bronchus just below the tracheal bifurcation(B). Compared to those obtained 3 years previously (A), the lesions increased in size. Bronchoscopy revealed 5 nodular smooth surfaced protrusions on the ventral side of the right mainstem bronchus just below the tracheal bifurcation (C, D). The maximum size was 5 mm in diameter.

A 60‐year‐old man was referred to our hospital because of abnormal findings on chest CT. Three years previously, an elevated lesion was noted in the right mainstem bronchus on chest CT at an annual health checkup as shown in Figure 1A. He underwent close follow up, however after 3 years the lesion increased in size as shown in Figure 1B. FDG‐PET showed no accumulation in this lesion. Upper and lower gastrointestinal endoscopy performed as part of the evaluation revealed no abnormality. Although the patient was asymptomatic, he was referred to our hospital for further evaluation.

He had a history of hypertension and diabetes mellitus. He had no history of smoking. His vital signs and physical examination were within normal limit. Complete blood counts, liver function tests, kidney function tests, and sIL‐2R level at 282 U/mL were all normal. Autoantibodies were negative.

**FIGURE 2:**
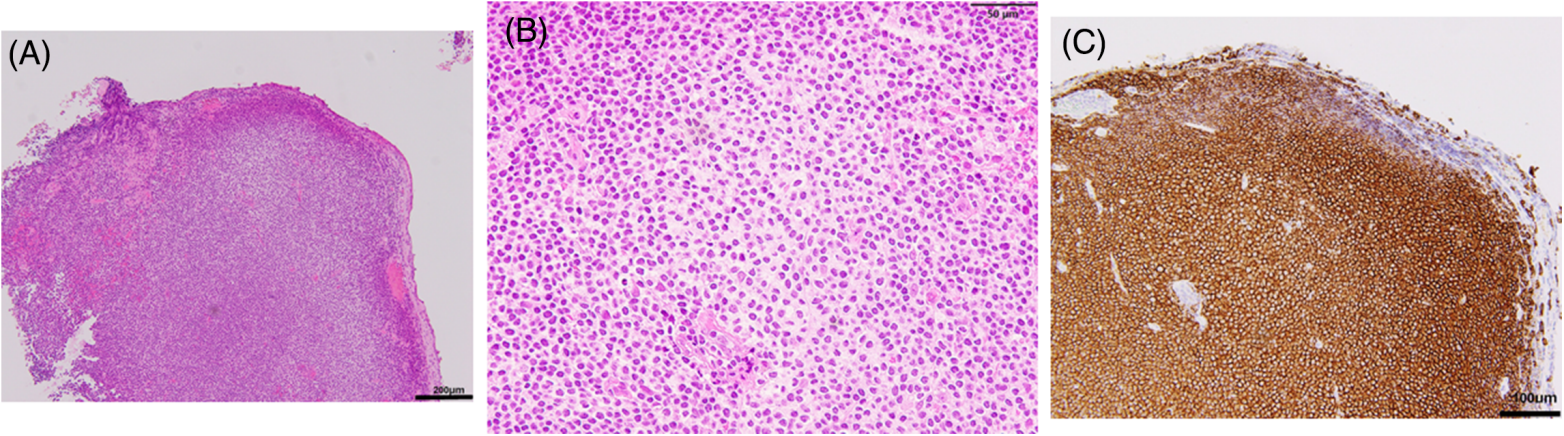
Transbronchial biopsy findings. An indistinct follicular structure (A, haematoxylin and eosin, ×100) and diffuse proliferation of atypical cells with distorted nuclei (B, haematoxylin and eosin, ×400) were observed. The infiltrating cells were diffusely positive for CD20 (C, CD20, ×200).

Bronchoscopy revealed 5 nodular smooth surface protrusions on the ventral side of the right main bronchus just below the tracheal bifurcation, with the largest lesion that measured 5 mm in diameter (Figure 1C,D). The histopathological findings indicate an indistinct follicular structure in low magnification (Figure 2A) and diffuse proliferation of atypical cells with distorted nuclei (Figure 2B). Immunostaining showed that the infiltrating cells were diffusely positive for CD20 (Figure 2C), and AE1&3 and CAM5.2 staining showed no clear lymphoepithelial lesions. Other stains were vimentin (+), CD3(−), chromogranin A (−), synaptophysin (−), CD56(−), TTF1(−), S100(−), p40(−), MIB1: 20%, Cyclin D1(−). Based on these findings, the diagnosis of MALT lymphoma was made.

Radiotherapy was instituted 15 times with 30 Gy in the area from the right main bronchus to the Botallo's lymph node. After the completion of radiotherapy, the elevated lesions in the right mainstem bronchus disappeared on follow up chest CT. After radiotherapy, the patient complained of mild discomfort (grade 1) during swallowing, which was thought to be caused by radiation esophagitis. The patient was placed under observation without treatment and the symptom completed abated after 1 month. The patient continues to undergo annual health check‐up including FDG‐PET, and to date no recurrence has been observed after 5 years.

## DISCUSSION

To the best of our knowledge, only 48 cases have been reported in the literature, including English‐language articles and 2 abstracts in English from conference presentations.^1^, ^2^, ^3^, ^4^, ^5^, ^6^, ^7^, ^8^, ^9^, ^10^, ^11^, ^12^, ^13^, ^14^, ^15^, ^16^, ^17^, ^18^, ^19^, ^20^, ^21^, ^22^, ^23^, ^24^, ^25^, ^26^, ^27^, ^28^, ^29^, ^30^, ^31^, ^32^, ^33^, ^34^, ^35^, ^36^, ^37^, ^38^ Furthermore, there has been only one report that reviewed the literature with 20 cases.^4^ We believe our review of 49 cases herein will provide valuable information and serve as a useful clinical reference for this rare disease. A summary of the reports including our case and those of autopsy cases, is shown in Table 1.

**TABLE 1:** Summary of the case reports.

No	Age(y)	Sex	Clinical symptom	Location	Endobronchial lesion pattern	Treatment	Follow‐up(M)	References
1	63	M	Cough, wheezing	Trachea, carina	Several nodular protrusions	Resection, RTx	ND	6
2	66	F	Dyspnea, stridor, cough	Trachea	Solitary intraluminal nodule	Resection, RTx	12	7
3	74	F	None	Trachea	Solitary intraluminal nodule	Laser photoresection	12	8
4	32	F	Dyspnea	Lt MB	Several nodular protrusions	CTx	36	10
5	44	F	Dyspnea	Trachea	Solitary intraluminal nodule	Resection	53	9
6	61	M	Cough	Rt BI	Solitary intraluminal nodule	RTx	48	2
7	82	M	None	Rt LLB	Diffuse wall thickening	ND	ND	1
8	46	M	Dyspnea	Lt LLB	ND	CTx	10	11
9	83	F	Cough, dyspnea, weight loss	Trachea, both MB	Several nodular protrusions	CTx	ND	12
10	70	F	Cough, dyspnea	Trachea, Rt MB	Several nodular protrusions	Cryo Tx, steroid therapy	18	13
11	44	F	None	Lt MB	Solitary intraluminal nodule	Sleeve resection, CTx	36	14
12	60	F	Dyspnea, cough, wheezing, hoarseness	Trachea	Solitary intraluminal nodule	RTx, CTx	ND	15
13	71	F	Cough, dyspnea, hoarseness, weight loss	Trachea	Diffuse wall thickening	CTx	ND	16
14	54	F	Cough	Trachea, Rt MB	Diffuse wall thickening	CTx	10	3
15	44	F	Sputum	Trachea, both MB	Several nodular protrusions	CTx	25	3
16	48	F	Cough	Trachea	Solitary intraluminal nodule	RTx	42	3
17	21	F	Wheezing	Trachea	Solitary intraluminal nodule	Cryo Tx	5	3
18	57	F	Hemoptysis	Lt LLB	Solitary intraluminal nodule	None	48	3
19	58	F	Dyspnea	Lt ULB	Solitary intraluminal nodule	RTx	36	3
20	61	M	Hemoptysis	trachea	Several nodular protrusions	RTx	1	3
21	49	F	Dyspnea	Trachea, Lt MB	Several nodular protrusions	Stent, CTx	12	17
22	61	M	ND	Trachea, Lt MB	Several nodular protrusions	CTx	ND	5
23	82	M	Dysphagia, cough	Trachea	Diffuse wall thickening	CTx	ND	5
24	73	M	None	Trachea	Several nodular protrusions	CTx	24	18
25	93	F	Wheezing	Trachea, Lt MB	Several nodular protrusions	Steroid therapy	5	19
26	37	F	Cough, blood‐streaked sputum, dysphonia	Trachea,	Solitary intraluminal nodule	CTx	3	20
27	46	F	Cough	Trachea, both MB	Diffuse wall thickening	ND	ND	21
28	64	M	None	Trachea	Several nodular protrusions	RTx	ND	22
29	50	M	Cough, chest pain, wheezing, hot flash	Carina, Rt MB, BI	Several nodular protrusions	CTx	12	23
30	54	F	Cough, dyspnea	Trachea	Several nodular protrusions	Cryo Tx	3	23
31	77	M	None	Rt LLB	Several nodular protrusions	None	19	4
32	62	M	None	Carina	Several nodular protrusions	RTx, CTx	25	4
33	47	F	Cough, dyspnea	Trachea, carina	Several nodular protrusions	RTx, steroid therapy	ND	24
34	61	F	Hemoptysis	Trachea	Several nodular protrusions	CTx	ND	24
35	57	M	Dyspnea	Trachea	Several nodular protrusions	RTx	15	25
36	73	M	Cough, dyspnea	Trachea, carina, both MB	Several nodular protrusions	None	12	26
37	70	M	Cough	Rt MLB	Several nodular protrusions	Sleeve lobectomy	ND	27
38	70	M	Cough, blood‐streaked sputum	Trachea	Several nodular protrusions	Resection	2	28
39	75	M	None	Carina	Solitary intraluminal nodule	None	6	29
40	81	F	Cough, dyspnea	Trachea	Several nodular protrusions	Resection	9	30
41	84	F	Cough, dyspnea	Lt MB	Several nodular protrusions	CTx	ND	31
42	48	M	Cough, myalgias, arthralgias, dyspnea	Lt ULB	Solitary intraluminal nodule	CTx	ND	32
43	72	M	Dyspnea	Trachea, Lt MB	Several nodular protrusions	RTx	ND	33
44	85	F	Cough	Lt ULB, LLB	Solitary intraluminal nodule	None	192	34
45	70	F	Weight loss	Rt ULB, MLB	Several nodular protrusions	CTx	ND	35
46	84	F	ND	Trachea	ND	HP eradication therapy	ND	36
47	61	F	Dyspnea, cough, stridor	Trachea	Several nodular protrusions	CTx	ND	37
48	55	M	Cough, sputum, chest stuffiness, dyspnea	Rt MB, Rt ULB	Several nodular protrusions	RTx	ND	38
49	60	M	None	Rt MB	Several nodular protrusions	RTx	54	Our case

Abbreviations: BI, bronchus intermedius; CryoTx, cryotherapy; CTx, chemotherapy; HP, *Helicobacter pylori*; LLB, lower lobar bronchus; Lt, left; MB, main bronchus; MLB, middle lobar bronchus; ND, no data; Rt, right; RTx, radiotherapy; ULB, upper lobar bronchus.

The initial symptoms and findings include cough, dyspnea, sputum and wheezing. A case of atelectasis because of progression of EML has been reported.^2^ The number of patients with such symptoms was 38 (77.6%), and 9 patients (18.4%) were found to be asymptomatic (no data available in 2 patients). Twenty‐one patients (42.9%) are male and 28 patients (57.1%) are female, indicating no significant gender difference. The location of the lesion in majority of the patients (39 patients, 79.6%) was within the central airway.

According to Yoon et al., intratracheal and endobronchial lesions in EML can be classified into the following three patterns: several nodular protrusions, solitary intraluminal nodule, and diffuse wall thickening.^3^ In the present study, this classification was adopted for analysis. As shown in Table 1, 57.1% had several nodular protrusions, 28.6% had solitary intraluminal nodules, and 10.2% had diffuse wall thickening (no data available in 2 patients). From these findings, it appears that multiple nodular protrusions or elevations in the trachea are an important finding suggestive of EML.^4^, ^5^

The standard treatment for EML has not been established yet. Out of the 49 patients, 20 patients (40.8%) received chemotherapy, with one undergoing stenting, two received radiotherapy, and one having surgery. Nine patients (18.4%) received radiotherapy alone. Other treatment options included observation, cryotherapy, Helicobacter pylori eradication, and systemic steroid therapy as symptomatic treatment. A patient who underwent Helicobacter pylori eradication treatment was judged to have progressive disease and was subsequently treated with chemotherapy. But none of the other 48 patients, experienced recurrence during the observation period ranging from 1 to 192 months. Chemotherapy regimen typically include cyclophosphamide, vincristine, prednisolone, plus rituximab (R‐COP) with addition of doxorubicin (R‐CHOP) if therapeutic response is inadequate.^11^ Furthermore in elderly patients, R‐COP may be more favourable due to concern of side effects with R‐CHOP.^12^ Chemotherapy regimen is continued until regression of tumour which typically requires 6–8 cycles.^5^, ^12^ To date, we have not identified a prospective report on the effect of chemotherapy alone, radiation therapy alone, or the combination of these two therapies. Increase of adverse effects associated with infection, neuropathy, and hematologic derangements poses a significant burden on patients. In our case, there was no recurrence for 60 months after radiotherapy. Although the patient complained of discomfort when swallowing, this was most likely due to radiation induced esophagitis which resolved without treatment with close follow up. Although only 18% of the cases received radiotherapy, it appears to be an effective mode of treatment and due to the limited number of adverse events associated with it, radiotherapy alone could potentially be considered for initial treatment modality for EML. Chemotherapy may be considered as a treatment algorithm when radiotherapy is not successful. Due to the small number of cases that have reported to date, further research with EML cases is needed.

Our case is unique because the lesion was first detected by annual health checkup including chest CT while the patient was asymptomatic. This allowed our case to be followed with FDG‐PET over the subsequent 3 years until the maximum diameter of the tumour reached 5 mm. From the time when there were no findings at all, we were able to follow up the course of the disease over a period of 4 years. The change in size of EML appeared to be variable from a report where the tumour size did not change over 16 years^34^ to another case that caused significant stenosis of the left mainstem bronchus.^10^ As for our case, the tumour size increased during subsequent annual follow ups that indicated a need for aggressive treatment measure. As reported in another case, EML is positive on FDG‐PET when tumour size reaches 11‐25 mm,^3^ which may explain why our case with a tumour size of 5 mm was not positive on FDG‐PET. There has been only one other case^4^ that was detected on chest CT during lung cancer screening. The other 7 cases that were incidentally found in asymptomatic patients occurred during examination for motor vehicle accident, follow up of both benign and malignant tumours in other organs, asbestos exposure, and so on.^1^, ^4^, ^8^, ^14^, ^18^, ^22^, ^29^

In conclusion, we report a case of EML which was found at an annual health checkup. Bronchoscopy revealed 5 nodular smooth surfaced protrusions on the ventral surface of the right mainstem bronchus. Biopsy revealed diffuse infiltration of small lymphocytes, positive for CD20, indicating MALT lymphoma. The lesions were successfully treated with radiotherapy, and no recurrence has been observed after 5 years. We reviewed 48 cases of EML in the literature and provided a comprehensive review of the literature to date including our case report. We believe our review of 49 cases will serve as an important clinical guide for clinicians that may encounter this very rare disease. Although the standard treatment for EML is not been established, radiotherapy appears to be effective with limited adverse events associated with treatment and therefore it can be potentially considered as the initial treatment for EML.

## AUTHOR CONTRIBUTIONS

Conception or design of the work, the acquisition, analysis or interpretation of data for the work: R.A., M.T., A.N., A.E., T.I., M.O., I.K. Drafting the work or reviewing it critically for important intellectual content: S.O., M.A., H.S., I.K. Final approval of the version to be published: R.A., M.T., A.N., A.E., T.I. S.O., M.A., M.O., H.S., I.K.

## CONFLICT OF INTEREST STATEMENT

None declared.

## ETHICS STATEMENT

The authors declare that appropriate written informed consent was obtained for the publication of this manuscript and accompanying images.

## Data Availability

The data that support the findings of this study are available on request from the corresponding author. The data are not publicly available due to privacy or ethical restrictions.
